# Supplementary material for: Hyperpolyploidization of hepatocyte initiates preneoplastic lesion formation in the liver
Source: Nat Commun. 2021 Jan 28;12:645. doi: 10.1038/s41467-020-20572-8 (PMC7844417; doi:10.1038/s41467-020-20572-8)
Supplement: Supplementary file 1 — Supplementary Information [file 41467_2020_20572_MOESM1_ESM.pdf]

Title

Hyperpolyploidization of hepatocyte initiates preneoplastic lesion formation in the liver

Authors

Heng Lin[1,2,11], Yen-Sung Huang[3,4,11], Jean-Michel Fustin[5,6], Masao Doi[7], Huatao Chen[8,9], Hui-Huang Lai[10], Shu-Hui Lin[1,2], Yen-Lurk Lee[3], Pei-Chih King[1,2], Hsien-San Hou[3], Hao-Wen Chen[1,2], Pei-Yun Young[1,2], Hsu-Wen Chao[1,2,11,] ✉

Affiliations

[1] Department of Physiology, School of Medicine, College of Medicine, Taipei Medical University, Taipei 11031, Taiwan

[2] Graduate Institute of Medical Sciences, College of Medicine, Taipei Medical University, Taipei 11031, Taiwan

[3] The Ph.D. Program for Translational Medicine, College of Medical Science and Technology, Taipei Medical University, Taipei, 11031, Taiwan

[4] Institute of Biomedical Sciences, Academia Sinica, Taipei 11529, Taiwan.

[5] Laboratory of Molecular Metabology, Graduate School of Pharmaceutical Sciences, Kyoto University, Sakyo-ku, Kyoto 606-8501, Japan.

[6] The University of Manchester, Faculty of Biology, Medicine and Health, Oxford Road, Manchester, M13 9PL, UK.

[7] Department of Systems Biology, Graduate School of Pharmaceutical Sciences, Kyoto University, Sakyō-ku, Kyoto 606-8501, Japan.

[8] Department of Clinical Veterinary Medicine, College of Veterinary Medicine, Northwest A&F University, Yangling, Shaanxi 712100, China

[9] Key Laboratory of Animal Biotechnology of the Ministry of Agriculture, Northwest A&F University, Yangling, Shaanxi 712100, China

[10] Department of Medical Laboratory Science and Biotechnology, College of Medicine,

National Cheng Kung University, Tainan 70101, Taiwan

[11] These authors contributed equally

✉Corresponding author: Hsu-Wen Chao, Taipei Medical University, 250 Wu-Hsing Street, Taipei city, Taiwan 110, E-mail: chaohw3619@tmu.edu.tw; Phone: +886-2-2789-9133

Funding

This work was supported by grants from the Taipei Medical University, Ministry of Science and Technology of Taiwan [MoST108-2628-B-038-002 and MoST106-2320-B-038-026].

Disclosure of Potential Conflicts Disclosure of Potential Conflicts

No potential conflicts of interest were disclosed.

Table of contents

Supplementary Figure 1..3
Supplementary Figure 2..8
Supplementary Figure 3..11
Supplementary Figure 4..13
Supplementary Figure 5..15
Supplementary Figure 6..17
Supplementary Figure 7..20
Supplementary Table 1..22

Supplementary Figures

Supplementary Figure 1

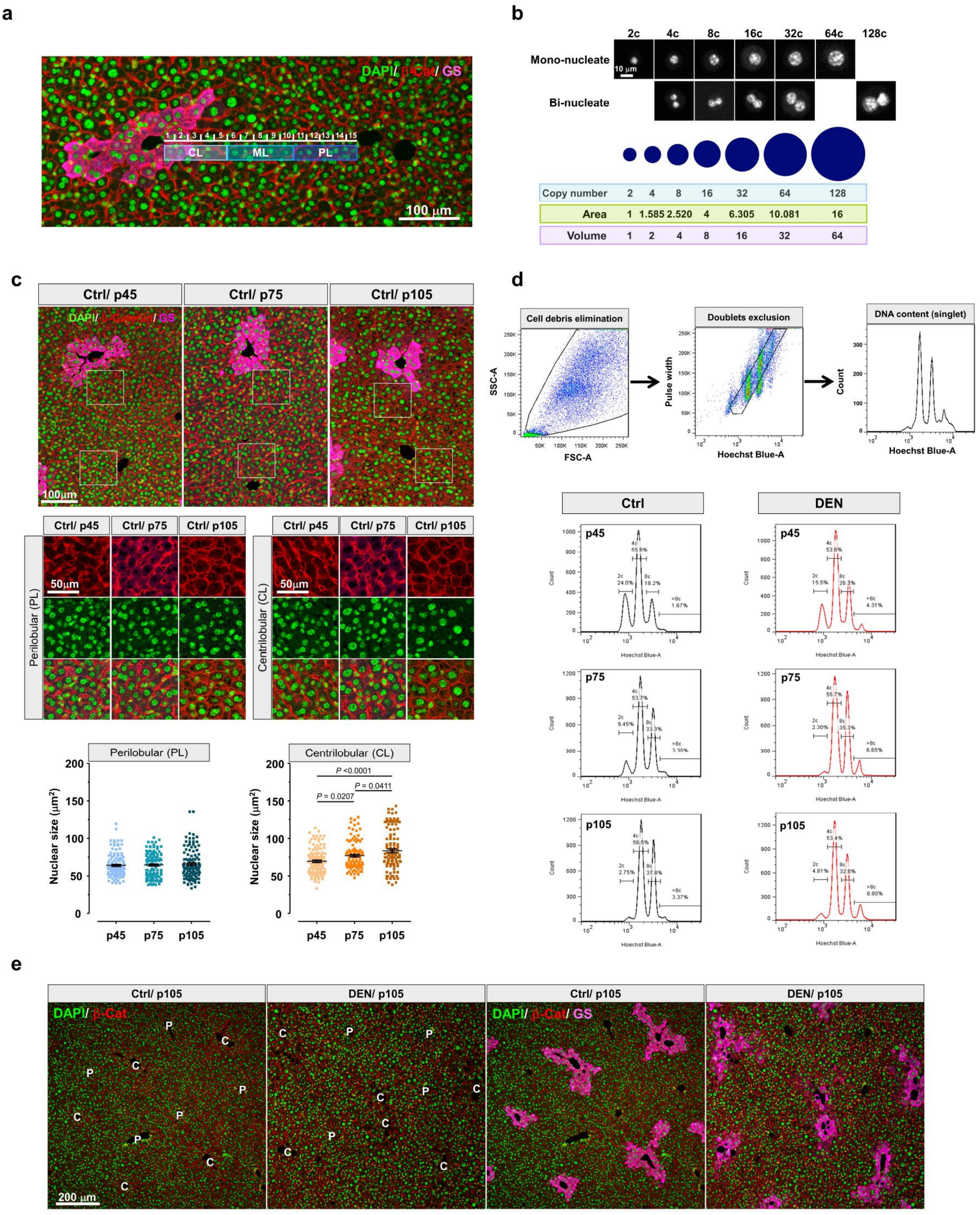

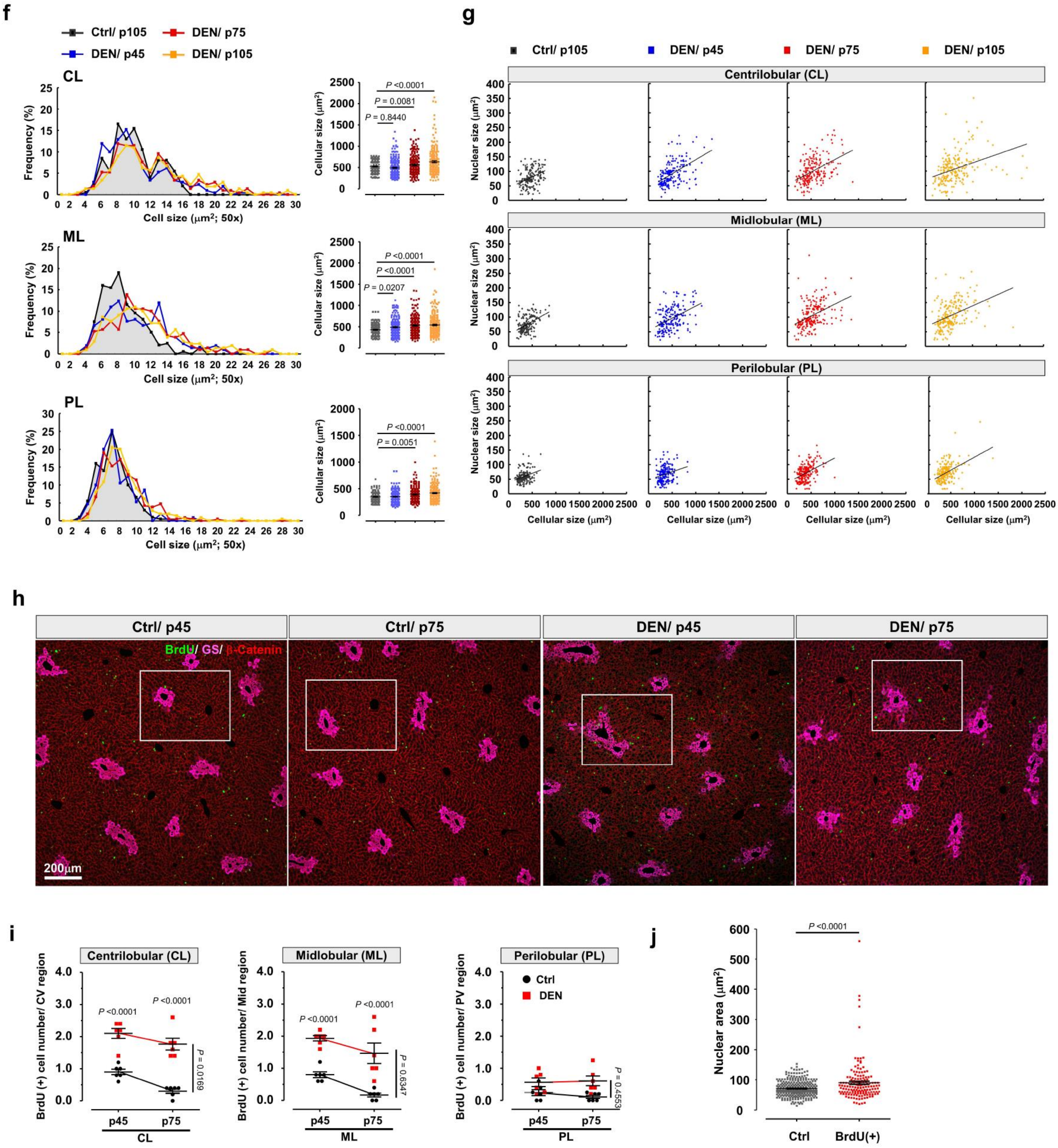

f
Ctrl/ p105
DEN/ p75
DEN/ p45
DEN/ p105
CL
ML
PL
Frequency (%)
Cell size (μm²; 50x)
Cellular size (μm²)
P <0.0001
P = 0.0081
P = 0.8440
P = 0.0207
P = 0.0051
g
Centrilobular (CL)
Midlobular (ML)
Perilobular (PL)
Nuclear size (μm²)
Cellular size (μm²)
h
Ctrl/ p45
Ctrl/ p75
DEN/ p45
DEN/ p75
BrdU/ GS/ β-Catenin
200μm
i
BrdU (+) cell number/ CV region
BrdU (+) cell number/ Mid region
BrdU (+) cell number/ PV region
P = 0.0169
P = 0.6347
P = 0.4553
Ctrl
DEN
p45
p75
CL
ML
PL
j
Nuclear area (μm²)
P <0.0001
Ctrl
BrdU(+)

k

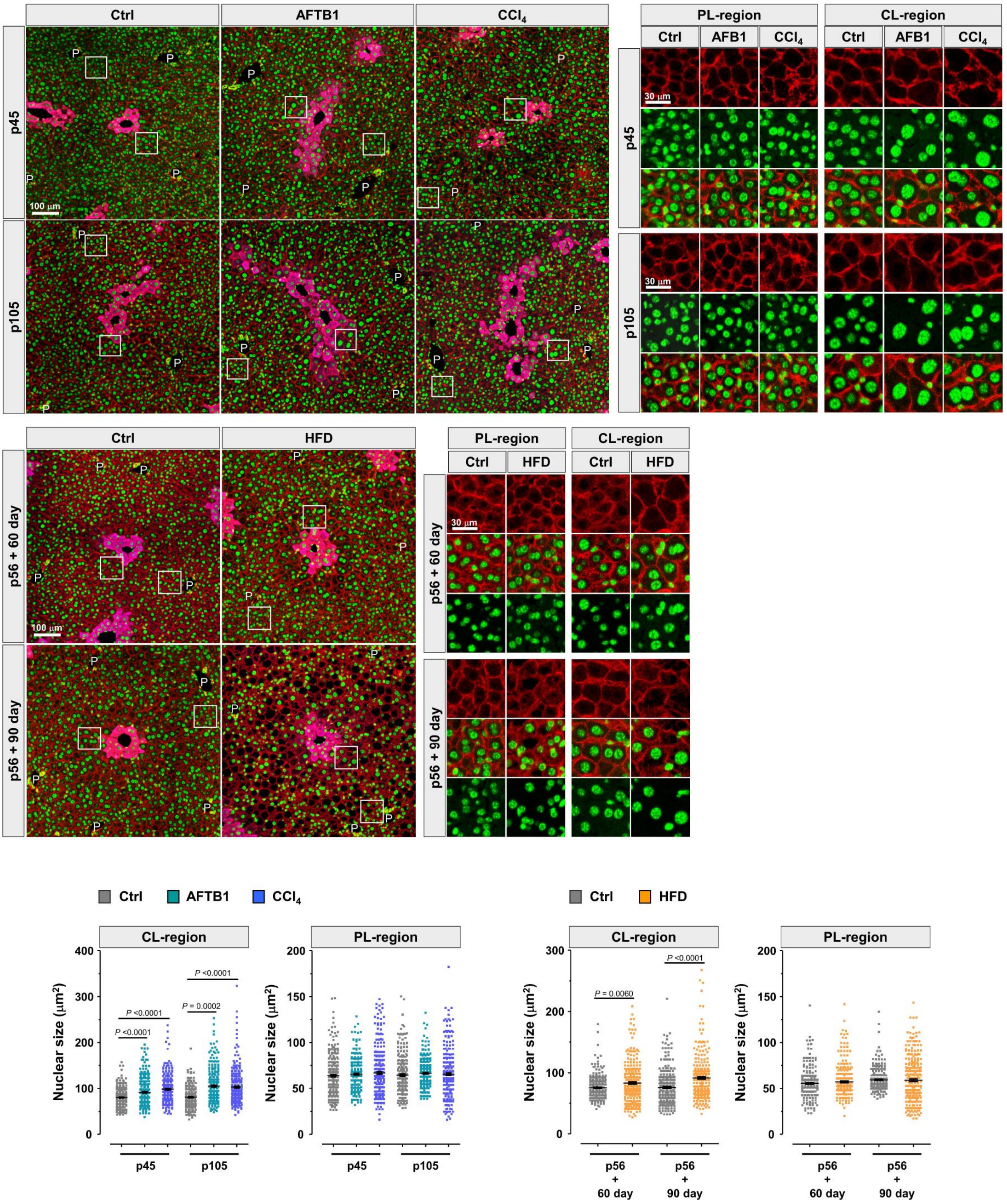
Ctrl
AFTB1
CCl_4
p45
p105
100 μm
P
PL-region
CL-region
Ctrl
AFB1
CCl_4
30 μm
Ctrl
HFD
p56 + 60 day
p56 + 90 day
Ctrl
AFTB1
CCl_4
CL-region
PL-region
Nuclear size (μm²)
P <0.0001
P <0.0001
P = 0.0002
P <0.0001
p45
p105
Ctrl
HFD
P = 0.0060
P <0.0001
p56 + 60 day
p56 + 90 day

Supplementary Figure 1. DEN treatment causes pathological hyperpolyploidy of hepatocytes within CL and ML regions. (**a**) Example of image displays subdividing of CV-PV axis into 15 parts for further analysis. Classic hexagonal shaped liver lobule with three specific zones including centrilobular (CL), midlobular (ML) and perilobular (PL) regions, and each specific zone is composed by 5 parts as indicated. Liver slice was co-stained with DAPI (green) and β-catenin (red), and CL regions marker GS (magenta). N = 5 mice in this examination. (**b**) Representative images show the nuclear morphology of hepatocytes isolated from three months DEN-treated mouse liver and counterstained with DAPI. Assuming that the concentration and distribution of nuclear chromatin are equal and similar in every hepatocyte, and it follows Lambert-Beer's law during image acquisition, the relationship between the cross-sectional area of the spherical nucleus and the ploidy can be shown as below. N = 3 mice in this examination. (**c**) Representative immunostaining of liver sections from control mice at indicated ages. High power images show the cell and nuclei size of hepatocytes within CL and PL region. Dot plot graphs illustrate the changes of cell and nuclei size of hepatocytes during liver development in control mice. Mouse number (N) = 3 per group, and cell number (n) = 120 per group. (**d**) Representative histograms of flow cytometry demonstrate the DAPI-labeled DNA content of control and DEN-treated hepatocytes at indicated ages. Cell debris were excluded on the flow cytometry profile based on the Forward Scatter (FSC-A) and Side Scatter area (SSC-A). Doublet and aggregated cells were gated out according to Hoechst Blue-A against Hoechst Blue-W. The DNA content of single hepatocyte was display as histograms which show the DNA copy numbers as the X-axis and cell numbers as Y-axis. DEN-treated hepatocytes showed more populations with higher DNA content than control hepatocytes as comparing at the same age. (**e**) Images of liver sections from control and three months DEN-treated liver under low magnification. The enlarged nuclei were observed in DEN-treated liver nearby CL and ML region of liver specifically. Liver slices were co-stained with DAPI (green) and β-catenin (red), and CL regions marker GS (magenta). (**f**) The quantitative data show the cell size of hepatocytes along CV-PV axis displayed as

frequency distribution and dot plot graphs. Mouse number (N) = 5 per group, and cell number (n) = 750 per group. (**g**) The scatter plot graph displays the relationship between nucleus and cell size in control and DEN-treated liver at indicated times. The bigger nucleus and cell size were detected in DEN-treated liver with time-dependent manner. Mouse number (N) = 5 per group, and cell number (n) = 750 per group. (**h**) Representative images show the distribution of BrdU in control and DEN-treated liver under lower magnification at indicated time. Boxed regions are shown in Fig. 1e with high magnification. Liver slices were co-stained with BrdU (green) and β-catenin (red), and CL regions marker GS (magenta). (**i**) Quantitative data display the numbers of BrdU positive hepatocytes at two developmental time points in control and DEN-treated livers. Higher numbers of BrdU positive hepatocytes were detected in DEN-treated liver within CL and ML region specifically but not PV region. N = 5 mice for each group. (**j**) Dot plot graph shows BrdU positive hepatocytes with the bigger nuclear area as compared with control groups. Cell number (n) = 300 for each group. (**k**) Immunostaining images of liver sections illustrate the cell and nucleus size of hepatocytes from age-matched control and drugs-treated livers including ATFB1, CCl_4, and 45 kcal% HFD at the indicated times. The nucleus and cell size were analyzed and showed as dot plot graph. Mouse number (N) = 3 per group, and cell number (n) = 300 per group. Statistic: One-way ANOVA with Bonferroni's post-test was used to (**c**), (**f**), and (**k**); Two-way ANOVA with Bonferroni's post-test was applied to (**i**). Two-tailed Student's unpaired t-test with Welch correction was used in (**j**). Values represent the mean ± SEM, Scale bars: 200 μm in (**e**) and (**h**), 100 μm in (**a**), (**c**), and (**k**). 50 μm in high power field of (**c**). 30 μm in high magnification of (**k**), 10 μm in (**b**).

Supplementary Figure 2

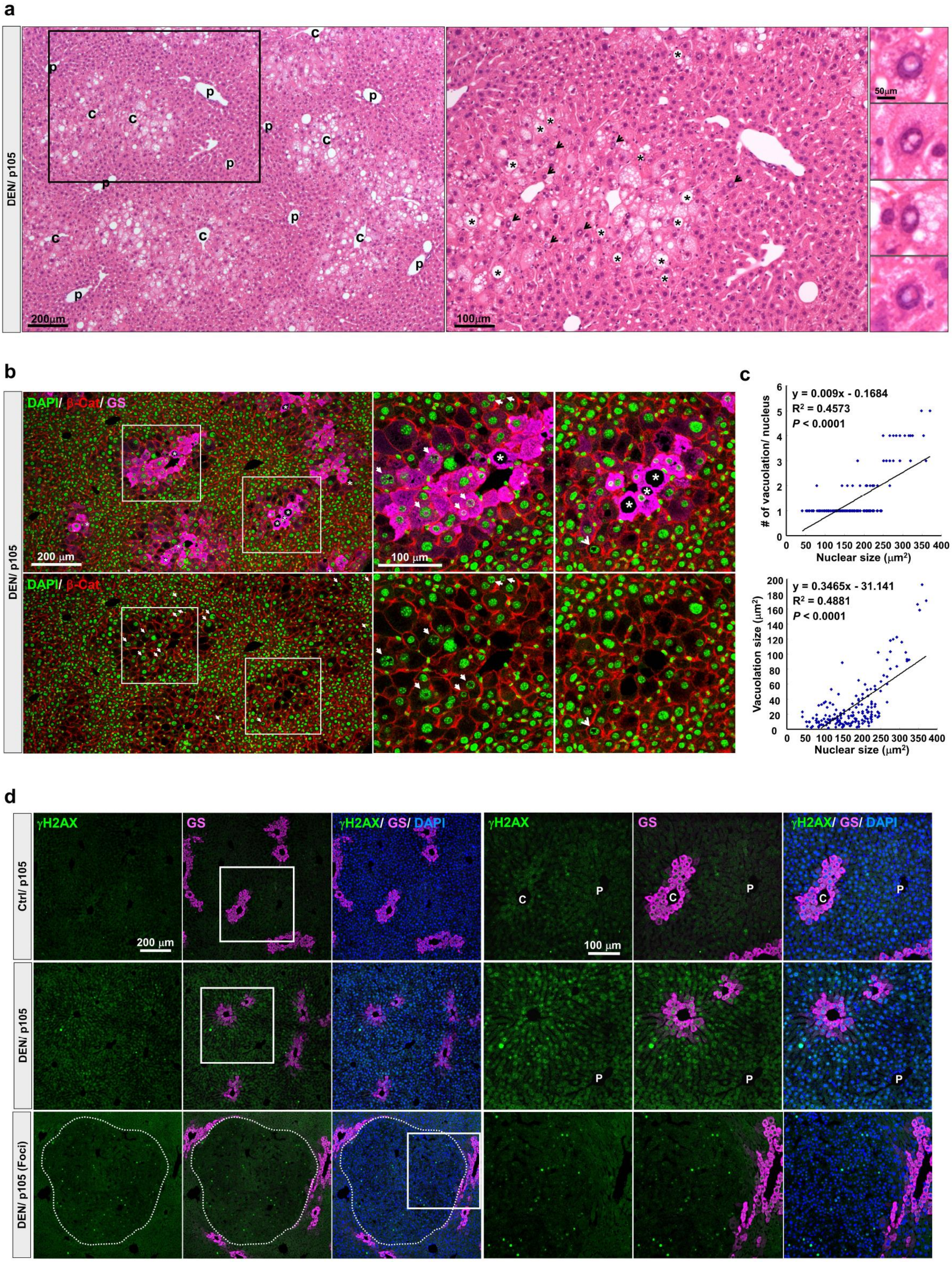

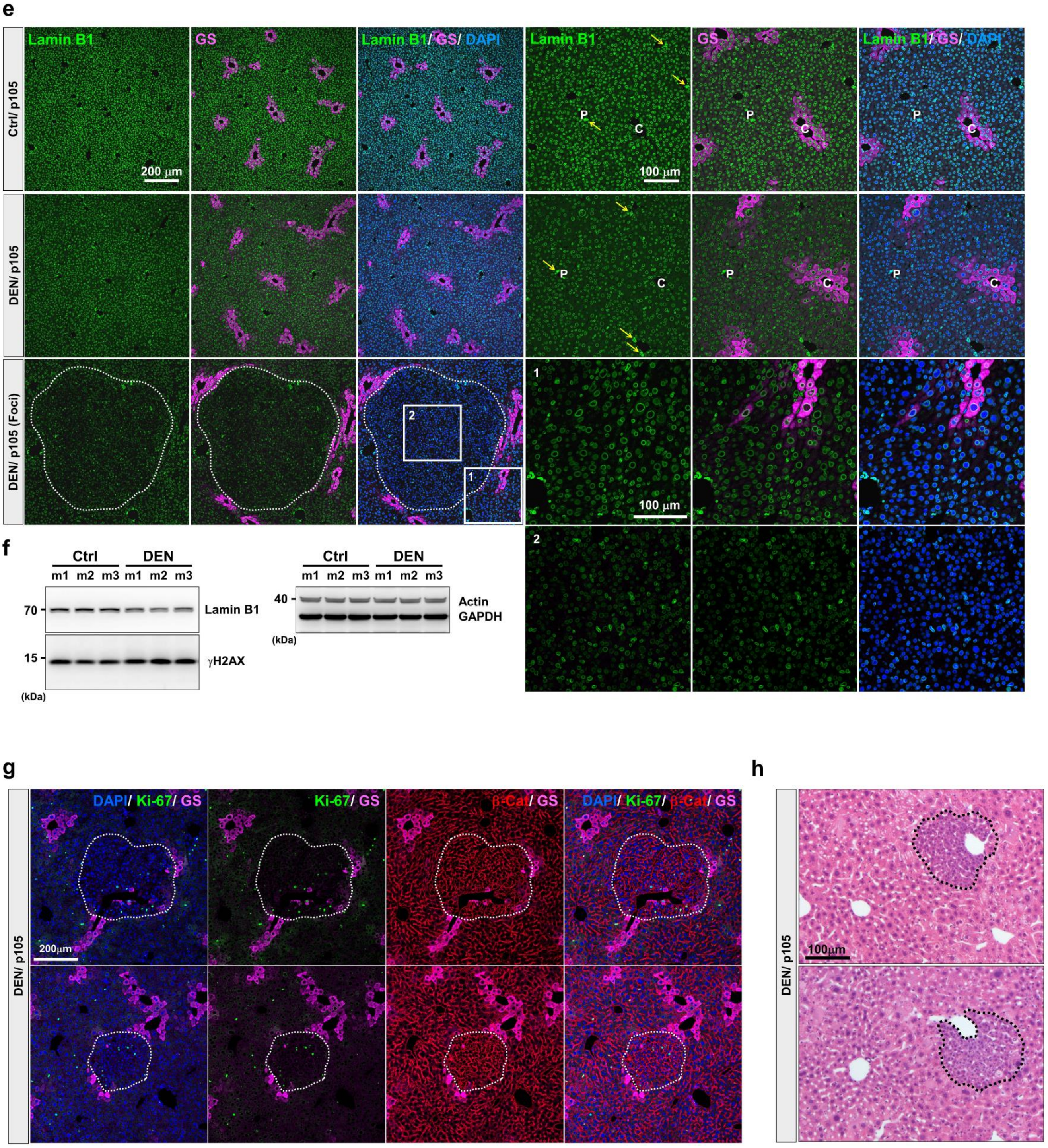

Supplementary Figure 2. DEN treatment causes appearance of preneoplastic lesions within CL and ML region. (**a**) Images of three months DEN-treated liver with H&E staining show the nuclear (arrow) and cytoplasmic (asterisk) vacuolation dominantly within CL and ML region of liver. High magnification displays the detail structure of nuclear and cytoplasmic vacuolation, which are located nearby CL and ML region. N = 5 mice in this treatment. (**b**) Nuclear and cellular morphology of three months DEN-treated livers were outlined by staining with DAPI

(green) and β-catenin (red), and GS (magenta). The high magnification images show that nuclear (arrow) and cytoplasmic (asterisk) vacuolation are detected in GS-positive hepatocytes, and GS signal were observed within the vacuolation region of nucleus. N = 5 mice in this treatment. (**c**) The correlation of the nuclear vacuolation number and size to nuclear size of hepatocytes was analyzed by linear regression. Each point represents an individual case from a hepatocyte. n > 170 hepatocytes with nuclear vacuolation from 5 mice. (**d**) The liver sections from control and three months DEN-treated mice were immunostained for γH2AX (green) and GS (magenta). DNA content was visualized by DAPI signal (blue). Highly expressed γH2AX was observed in hepatocytes adjacent to CL region and preneoplastic foci (dashed line). N = 3 mice for each group. (**e**) Representative immunostaning images indicate the expression of Lamin B1 (green) in the liver. Nuclei and CL region were outlined by DAPI (Blue) and GS signal (magenta), respectively. Three months DEN-treated hepatocytes showed lower expression of Lamin B1 than those in control; and the lowest Lamin B1 signal was observed within preneoplastic foci. Note that Lamin B1 signal showed similar expression level in portal triad cells (yellow arrow) in all groups. P indicates portal vein region. N = 3 mice for each group. (**f**) Protein level of γH2AX and Lamin B1 in control and DEN-treated livers were examined by immunoblotting. N = 3 mice for each group. (**g**-**h**) Immunohistochemistry (**g**) and H&E staining (**h**) show the dominant distribution of preneoplastic foci (dashed circles) nearby GS positive hepatocytes and CL region of the liver. Ki-67 positive signal was enriched within preneoplastic foci. For immunohistochemistry, liver slices were co-stained with DAPI (blue), Ki-67 (green), β-catenin (red), and GS (magenta). N = 5 mice in this treatment. Linear regression was used to (**c**). Values represent the mean ± SEM, Scale bars: 200 μm in low magnification of (**a**), (**b**), (**d**), (**e**), and (**g**), 100 μm in high magnification of (**a**), (**b**), (**d**), (**e**), and (**h**), and 50 μm in nuclear vacuolation images of (**a**).

Supplementary Figure 3

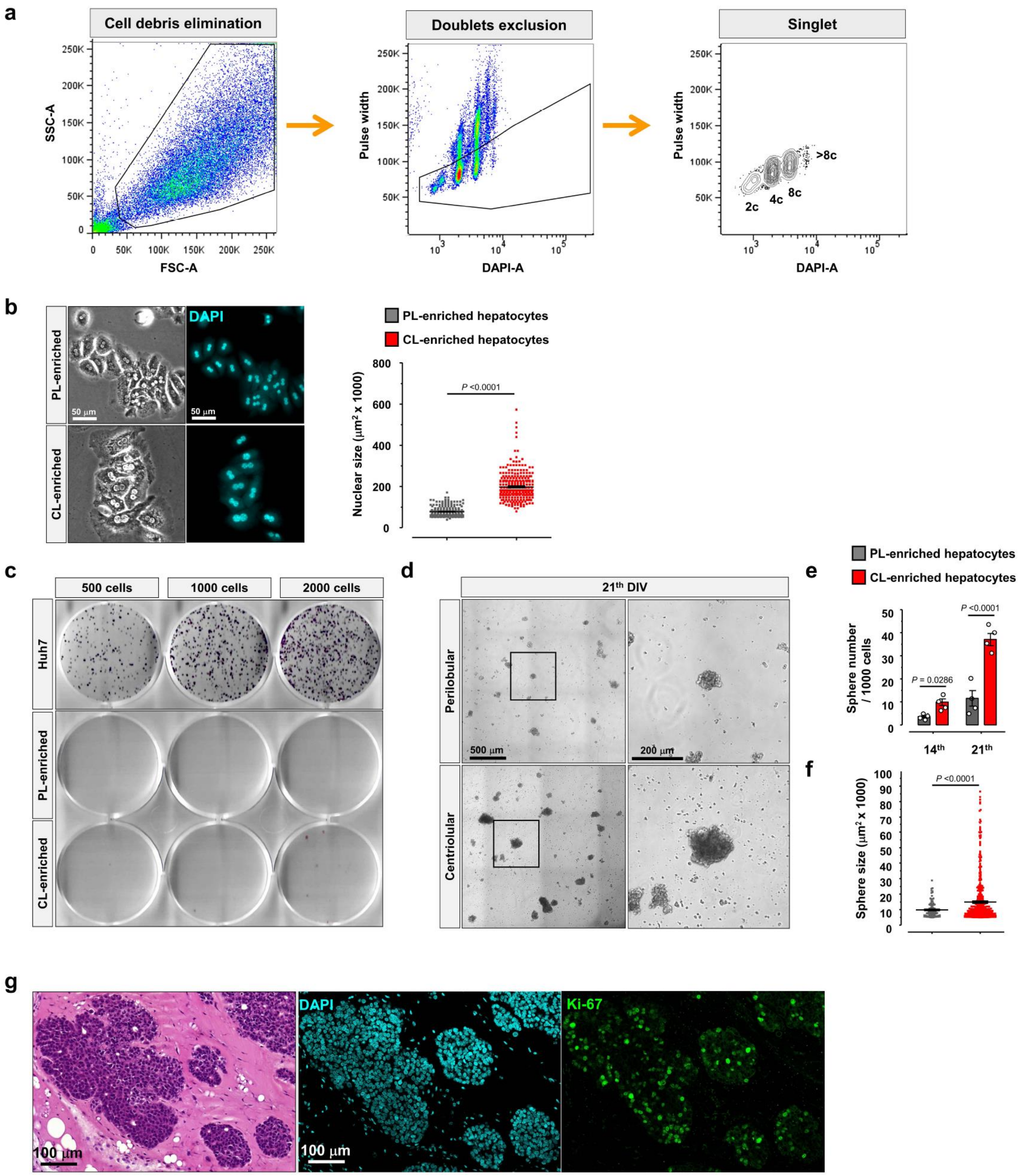

Supplementary Figure 3. CL-enriched hepatocytes show higher tumor stem cell characteristic. (**a**) Schematic illustration of gating strategy for the quantification of hepatocyte DNA content by flow cytometry after isolation through digitonin-collagenase infusion protocol from mouse livers. Abbreviation: FSC-A, forward scatter area; SSC-A, side scatter area. (**b**) Representative images display the cellular morphology and nucleus size of CL- or PL-enriched hepatocytes after 36h of seeding. Nuclear size was outlined by DAPI staining, and the quantitative data indicates that CV-enriched hepatocytes display larger nucleus size compared with PV-enriched hepatocytes. n = 300 hepatocytes/group. (**c**) Representative images show the colony formation assay with indicated cultured cell numbers in 6-well plate. Cells were fixed at 21th DIV and stained with crystal violet for colony numbers analysis, and Huh7 cells are the positive control. (**d**) Example of images show the tumorsphere formation of CL- and PL-enriched hepatocytes at 21th DIV. (**e-f**) The quantitative data indicates that tumorspheres derived from CL-enriched hepatocytes display higher numbers and larger size compared to PL-enriched group. (**g**) Representative immunohistochemistry and H&E stained images display the tumor cells derived from CL-enriched hepatocytes after three months of subcutaneous transplantation in NSG™ mice. Ki-67 signal positive cells (green) were enriched within the tumor nodule. DNA content was counter stained with DAPI (cyan). Statistic: Two-tailed Student's unpaired t-test with Welch correction was used in (**b**) and (**f**); Two-way ANOVA with Bonferroni's post-test was applied to sphere numbers of (**e**). Values represent the mean ± SEM, Scale bars: 500 μm in high magnification of (**d**), 200 μm in low magnification of (**d**), 100 μm in (**g**), and 50 μm in (**b**).

Supplementary Figure 4

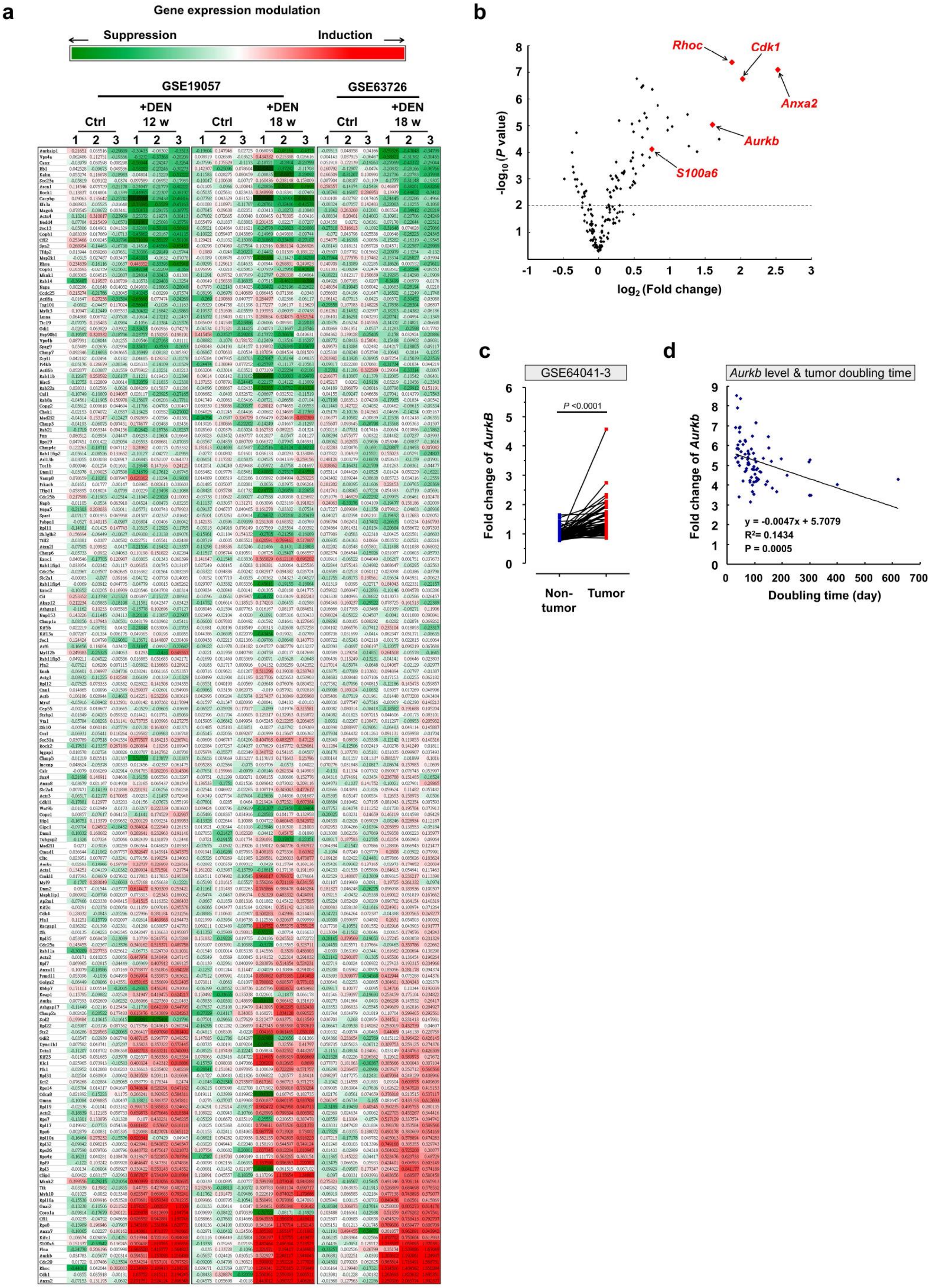

Supplementary Figure 4. *Aurkb* is a target for hyperpolyploidization of hepatocytes. (**a**) Heatmap of relative expression of cytokinesis genes up- or down-regulated by DEN in the liver. Cytokinesis genes were characterized and identified by previous studies[33-35], hepatic gene expression changes were obtained from two GEO datasets (GSE19057 and GSE63726)[36-37]. (**b**) Scatter plot graph with logarithmic and *P*-value axis shows the top five cytokinesis genes that are upregulated significantly in two GEO datasets. (**c**) Human *Aurkb* expression in matched normal and HCC biopsies, hepatic gene expression changes were obtained from two GEO datasets. Each pair data indicates one patient. N = 60 patients with HCC. (**d**) The scatter plot graph shows the linear regression analysis of the correlation between *Aurkb* expression and tumor doubling time of patient. Each point represents an individual case from a patient. N = 81 patients with HCC. Statistic: Two-tailed Student's paired t-test was used in (**c**). Linear regression was conducted to (**d**). Values represent the mean ± SEM,

Supplementary Figure 5

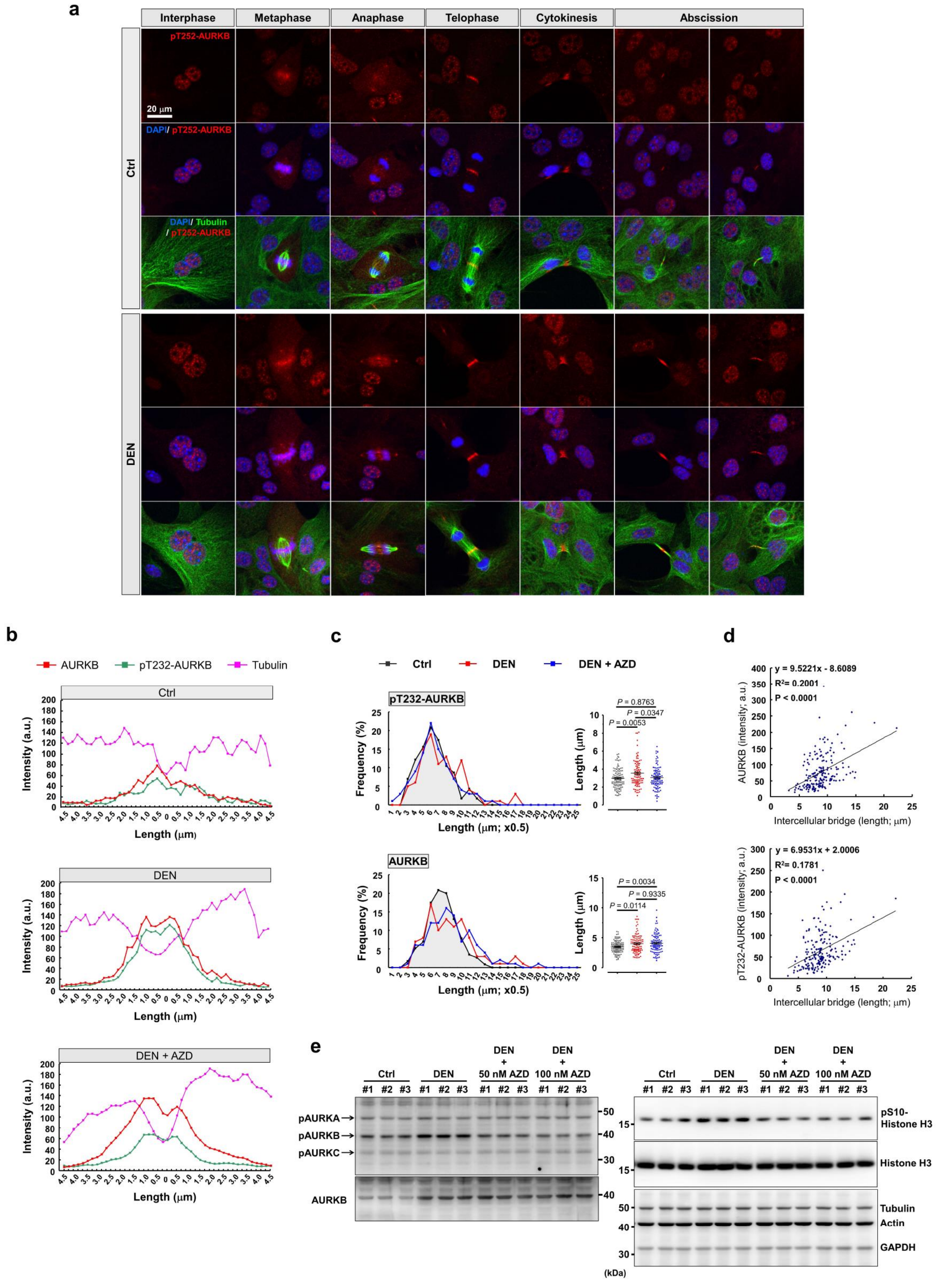

Supplementary Figure 5. Enrichment of pT232-AURKB at the midbody after DEN treatment. (**a**) Example of images show the immunofluorescence of pT232-AURKB (red) at different stage of cell cycle. Higher expression of pT232-AURKB was detected in hepatocytes isolated from liver with DEN treatment. Similar subcellular distribution of pT232-AURKB was observed between control and DEN-treated hepatocytes through whole cell cycle stages. Notable, highly enriched pT232-AURKB was expressed at the midbody during abscission stage in DEN-treated hepatocytes. The morphology of nuclei and intercellular bridges were outline by counterstaining with tubulin (green) and DAPI (blue) respectively. (**b**) Example of line-scan analysis of pT232-AURKB and AURKB intensity at the midbody (tubulin signal) from indicated cultured hepatocytes in Fig. 4h. (**c**) Frequency distribution and dot plot graphs show the length of AURKB and pT232-AURKB signal along the intercellular bridge. Longer AURKB and pT232-AURKB signal were detected in DEN-treated dividing hepatocytes compare to control group, that is rescued by 50 nM AZD1152 treatment. n > 100 dividing hepatocytes at abscission stage per group from three independent experiments. (**d**) Linear regression analysis shows the correlation between the expression level of AURKB and pT232-AURKB at the midbody and intercellular bridge length. n > 100 dividing hepatocytes at abscission stage from three independent experiments. (**e**) Immunoblots display that phosphorylation of AURKB at Thr232 residue and the downstream target signal of AURKB were inhibited by AZD1152 treatment. Statistic: One-way ANOVA with Bonferroni's post-test was applied to (**c**). Linear regression was conducted to (**d**). Values represent the mean ± SEM, Scale bars: 20 μm in (**a**).

Supplementary Figure 6

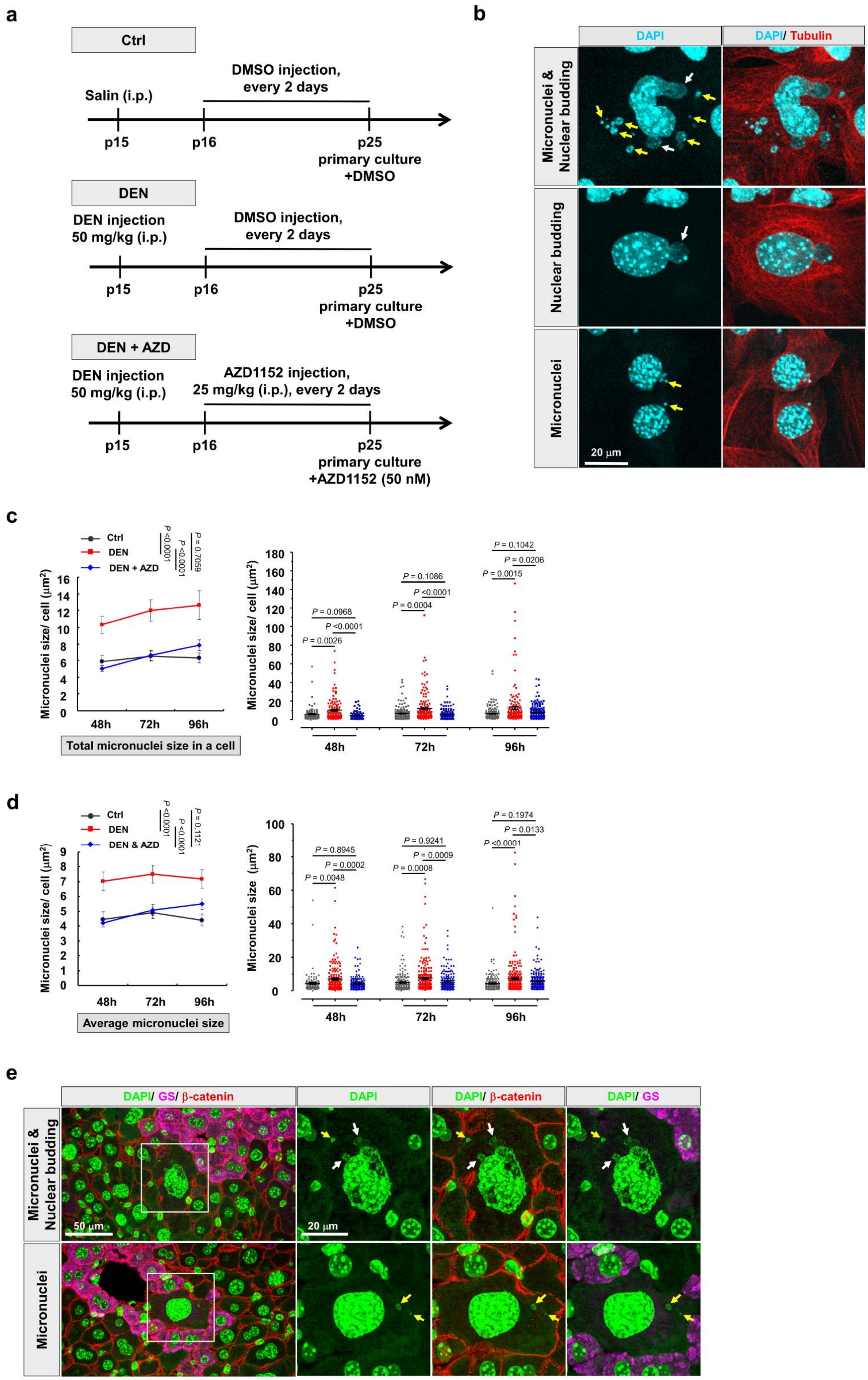

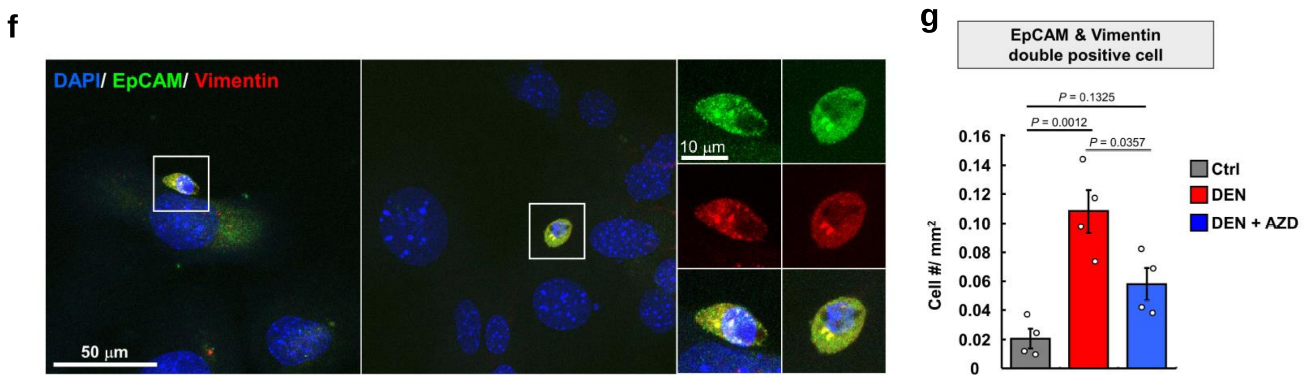

Supplementary Figure 6. Analysis the properties of nuclear budding and micronuclei in drugs treated cultured hepatocytes. (**a**) Schematic illustration of the hepatocyte primary culture and drugs administration protocol. Mice were injected with saline or DEN at p15 and sacrifice at p25. One day after DEN injection, AZD1152 (25 mg/kg) was applied to DEN-treated mice with every two days, while control and DEN only mice were treated with the same concentration of DMSO in saline. (**b**) Representative images show the typical morphology of micronuclei (yellow arrow) and nuclear budding (white arrow) in hepatocytes with DEN treatment. A variety of sizes and shapes of micronuclei and nuclear budding were observed. Micronuclei and nuclear budding have been defined as structures that are responsible for the expulsion of undesirable DNA content. Nuclear budding had a connection with the main nucleus, some were circular and positioned close to the nucleus, while some resided in the cytoplasm separated from the nucleus called micronuclei. (**c** and **d**) Total and average micronuclei size in a cell were analyzed showing a significant increase in DEN-treated hepatocyte at indicated time points. Note that the size was dramatically reduced after AZD1152 treatment in DEN-treated group. n > 100 cells with micronuclei from three independent experiments. (**e**) Example of liver slice images displays micronuclei (yellow arrow) and nuclear budding (white arrow) within hyperpolyploid hepatocytes in the liver with 3 months of DEN treatment. N = 5 mice in this treatment. (**f-g**) Immunocytochemistry shows example images of double staining cancer stem cell markers, EpCAM and Vimentin, in cultured hepatocytes isolated from DEN-injected liver after one week culture. Highly expression of both

EpCAM and Vimentin was observed in cells with smaller nuclei which is adjacent to the hepatocytes with bigger nucleus size. Significantly increased the numbers of EpCAM and Vimentin double positive cell in cultured hepatocytes isolated from the DEN-treated liver. AZD treatment dramatically reduced the number of EpCAM and Vimentin double positive cell in DEN-treated hepatocytes. Statistic: Two-way ANOVA with Bonferroni's post-test was applied to line graphs of (**c**), and (**d**); One-way ANOVA with Bonferroni's post-test was used to dot plot graphs of (**c**), (**d**), and (**g**). Values represent the mean ± SEM, Scale bars: 50 μm in low magnification of (**e**) and (**f**), 20 μm in (**b**) and high magnification of (**e**), 10 μm in high magnification of (**f**).

Supplementary Figure 7

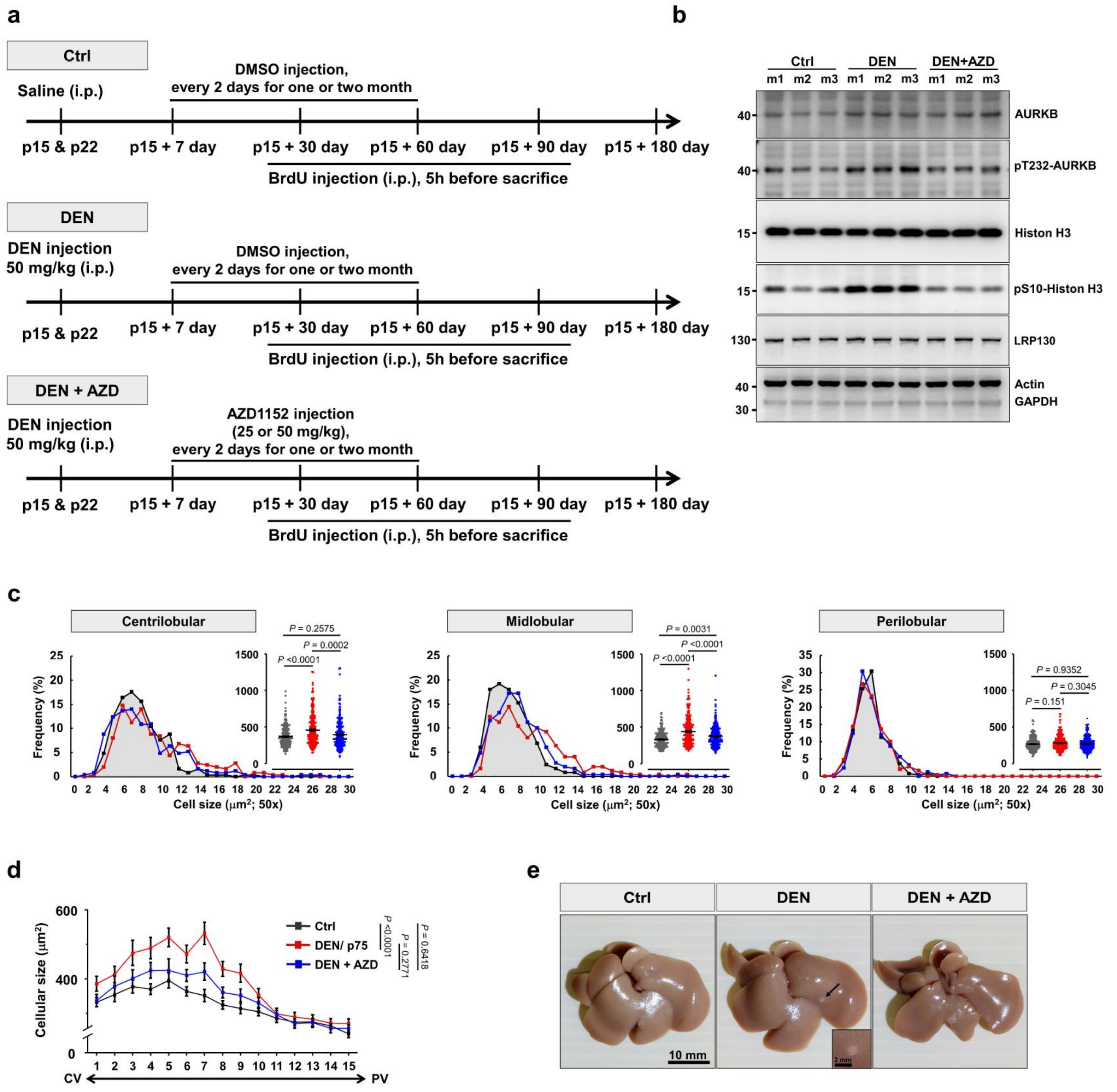

Supplementary Figure 7. Inhibition of AURKB activity reduces cell size of hepatocytes in DEN-treated liver. (**a**) Schematic illustration of drugs administration protocol in mice. Mice were injected with saline or DEN twice at p15 and p22. One week after DEN injection, AZD1152 was applied to DEN-treated mice every two days for one or two months, while control and DEN only group were treated with the same concentration of DMSO in saline. (**b**) Western blots show the significant decrease of pT232-AURKB in DEN-treated liver after one month of AZD1152 (25 mg/kg) injection. Phosphorylation of histone H3 at Ser10 residue,

downstream signal of AURKB, were reduced in one month of AZD1152 treated livers. Protein loading equivalence was controlled by b-actin, GAPDH, and LRP130 levels, which stands for the internal control at different molecule size. (**c**) Frequency distribution and dot plot graphs show the quantitative data of cell size of hepatocytes within specific liver lobule in indicated mice. N = 5 mice per group, and n = 750 hepatocytes per group. (**d**) Frequency distribution of cellular size of hepatocytes along CV-PV axis in indicated mice. N = 5 mice and n = 750 hepatocytes for each group. (**e**) Example of liver images display the global morphology livers with or without drugs treatment. The general morphology shows no significant change between control and DEN-treated liver, although very few tumor nodules were found on the surface of DEN-treated liver infrequently. No tumor nodules were discovered on the surface of DEN-treated liver followed AZD1152 (25 mg/kg) treatment. Statistic: One-way ANOVA with Bonferroni's post-test was used to (**c**); Two-way ANOVA with Bonferroni's post-test was applied to (**d**). Values represent the mean ± SEM, Scale bars: 10 mm in (**e**).

Supplementary Table 1

Table 1. Primer pairs utilized in qPCR.

Oligo title	Sequence (5' to 3')	UPL probe #
Anxa2-f	ggaaatatggcaagtccctgt	#42
Anxa2-r	tctggtagtcacccttggtgt	
Aurkb-f	attgcagactttggctggtc	#69
Aurkb-r	aatcatctctgggggcagat	
Cdk1-f	gaacttcgacatccaaatatagtcag	#64
Cdk1-r	ccatggacaggaactcaaaga	
Rhoc-f	aaggacctgaggcaagatga	#92
Rhoc-r	aaggcactgatcctgtttgc	
S100a6-f	aggaaggtgacaagcacacc	#17
S100a6-r	agcatcctgcagcttgga	
GS-f	gagcccaagtgtgtggaag	#58
GS-r	aaggggtctcgaaacatgg	
Axin2-f	ttattgctactccaaatgcaaaag	#50
Axin2-r	ttttggcaaggtaccacctc	
Rhbg-f	tcacactggtgtttgcctct	#18
Rhbg-r	gaagcattgggagtctggag	
Oat-f	taacgatctgcccgcact	#22
Oat-r	aacgataacgcctgcttcac	
Lect2-f	gcaccattcactgggaagata	#02
Lect2-r	tgtagaaaattttgacacaaaaacct	
Pck1-f	ggagtacccattgagggtatcat	#49
Pck1-r	gctgagggcttcatagacaag	
Gls2-f	tgacttctcgggccagttt	#88
Gls2-r	gcccatgacattgggtaca	
Arg1-f	cctgaaggaactgaaaggaaag	#02
Arg1-r	ttggcagatatgcagggagt	
Cps1-f	ccctctgactatgttgccatt	#72
Cps1-r	gggtcagcatctctcagtcg	
Cyp2f2-f	aaataccccaaagtgcaagc	#11
Cyp2f2-r	tgcatctgtgtaaggcatgg	
Actb-f	ctaaggccaaccgtgaaaag	#64
Actb-r	accagaggcatacagggaca	
Gapdh-f	gggttcctataaatacggactgc	#52
Gapdh-r	ccattttgtctacgggacga	
