# Supplementary material for: Hyperpolyploidization of hepatocyte initiates preneoplastic lesion formation in the liver
Source: Nat Commun. 2021 Jan 28;12:645. doi: 10.1038/s41467-020-20572-8 (PMC7844417; doi:10.1038/s41467-020-20572-8)
Supplement: Supplementary file 3 — Description of Additional Supplementary Files [file 41467_2020_20572_MOESM3_ESM.pdf]

**Description of Additional Supplementary Files**

File Name: Supplementary Movie 1
Description: Hepatocyte performed complete cytokinesis and became two individual daughter cells with single nucleus. Cyan arrow indicating the location of nucleus.

File Name: Supplementary Movie 2
Description: Hepatocyte performed cytokinesis failure without contractile ring formation and became one binucleate hepatocyte. Cyan arrow indicating the location of nucleus.

File Name: Supplementary Movie 3
Description: Hepatocyte performed abscission failure and became one binucleate hepatocyte. Cyan arrow indicating the location of nucleus.

File Name: Supplementary Movie 4
Description: Hepatocyte performed twice abscission failure and became one binucleate hepatocyte with enlarged nuclei. Cyan and yellow arrows indicating the location of nuclei and cleavage furrow, respectively.

File Name: Supplementary Movie 5
Description: Hepatocyte performed abscission failure followed by a complete cytokinesis and became two mononucleate hepatocytes with an enlarged nucleus. Cyan and red arrows indicating the location of nuclei and cellular boundary between daughter cells, respectively.

File Name: Supplementary Movie 6
Description: Colony formation of CL hepatocytes isolated from DEN-treated liver.

File Name: Supplementary Movie 7
Description: Colony formation of PL hepatocytes isolated from DEN-treated liver.
