# Supplementary material for: Hyperpolyploidization of hepatocyte initiates preneoplastic lesion formation in the liver
Source: Nat Commun. 2021 Jan 28;12:645. doi: 10.1038/s41467-020-20572-8 (PMC7844417; doi:10.1038/s41467-020-20572-8)
Supplement: Supplementary file 11 — Reporting Summary [file 41467_2020_20572_MOESM11_ESM.pdf]

nature research

Corresponding author(s): Hsu-Wen Chao

Last updated by author(s): 2020/11/09

Reporting Summary

Nature Research wishes to improve the reproducibility of the work that we publish. This form provides structure for consistency and transparency in reporting. For further information on Nature Research policies, see our Editorial Policies and the Editorial Policy Checklist.

Statistics

For all statistical analyses, confirm that the following items are present in the figure legend, table legend, main text, or Methods section.

n/a	Confirmed	
☐	☒	The exact sample size (*n*) for each experimental group/condition, given as a discrete number and unit of measurement
☐	☒	A statement on whether measurements were taken from distinct samples or whether the same sample was measured repeatedly
☐	☒	The statistical test(s) used AND whether they are one- or two-sided<br>*Only common tests should be described solely by name; describe more complex techniques in the Methods section.*
☒	☐	A description of all covariates tested
☐	☒	A description of any assumptions or corrections, such as tests of normality and adjustment for multiple comparisons
☐	☒	A full description of the statistical parameters including central tendency (e.g. means) or other basic estimates (e.g. regression coefficient) AND variation (e.g. standard deviation) or associated estimates of uncertainty (e.g. confidence intervals)
☐	☒	For null hypothesis testing, the test statistic (e.g. *F*, *t*, *r*) with confidence intervals, effect sizes, degrees of freedom and *P* value noted<br>*Give P values as exact values whenever suitable.*
☒	☐	For Bayesian analysis, information on the choice of priors and Markov chain Monte Carlo settings
☐	☒	For hierarchical and complex designs, identification of the appropriate level for tests and full reporting of outcomes
☒	☐	Estimates of effect sizes (e.g. Cohen's *d*, Pearson's *r*), indicating how they were calculated

Our web collection on statistics for biologists contains articles on many of the points above.

Software and code

Policy information about availability of computer code

Data collection	Quantitative PCR was performed by Lightcycler 480 system (Roche).<br>Enhanced chemiluminescence was detected by Image Quant LAS 4000 (Fujifilm).<br>Acquired data of flow cytometry were analyzed by BD FACSDiva software v6.2.<br>Confocal images were acquired by Carl Zeiss, 2011 SP7 FP3, version 14.0.0.0.<br>MetaMorph 7.6.5.0 was used for time-lapse recording.<br>FlowJo 7.6.1, Engine 2.79000 OS version was used for flow cytometry analysis.
Data analysis	GraphPad Prism version 8.4.0 (671) was used for statistic analysis.<br>Images were analyzed by using ImageJ 1.51j8.

For manuscripts utilizing custom algorithms or software that are central to the research but not yet described in published literature, software must be made available to editors and reviewers. We strongly encourage code deposition in a community repository (e.g. GitHub). See the Nature Research guidelines for submitting code & software for further information.

Data

Policy information about availability of data

All manuscripts must include a data availability statement. This statement should provide the following information, where applicable:

- Accession codes, unique identifiers, or web links for publicly available datasets
- A list of figures that have associated raw data
- A description of any restrictions on data availability

Source data are available as a Source Data file. The source data underlying Figures 1d, 1f-1i, 2b-2e, 2h-2i, 3a, 3e, 3g, 3i-3j, 3l, 4c-4h, 5c-5e, 6b-6c, 6e-6f, 7b, 7d-g, 7i, Supplementary Figures 1c, 1f, 1i-1j, 1k, 2c, 2f, 3b, 3e-3f, 4a-4d, 5b-5e, 6c-6d, 6g, 7b-7d are provided as a Source Data file. All the other data supporting the findings

of this study are available within the article and its supplementary information files and from the corresponding author upon reasonable request. A reporting summary and Supplementary Figures for this article are available as a Supplementary Information file. NCBI GEO datasets are available as follows
GSE19057 (https://www.ncbi.nlm.nih.gov/geo/query/acc.cgi?acc=GSE19057)
GSE63726 (https://www.ncbi.nlm.nih.gov/geo/query/acc.cgi?acc=GSE63726)
GSE89632 (https://www.ncbi.nlm.nih.gov/geo/query/acc.cgi?acc=GSE89632)
GSE66232 (https://www.ncbi.nlm.nih.gov/geo/query/acc.cgi?acc=GSE66232)
GSE20140 (https://www.ncbi.nlm.nih.gov/geo/query/acc.cgi?acc=GSE20140)
GSE54236 (https://www.ncbi.nlm.nih.gov/geo/query/acc.cgi?acc=GSE54236)
GSE64041 (https://www.ncbi.nlm.nih.gov/geo/query/acc.cgi?acc=GSE64041

Field-specific reporting

Please select the one below that is the best fit for your research. If you are not sure, read the appropriate sections before making your selection.

☒ Life sciences ☐ Behavioural & social sciences ☐ Ecological, evolutionary & environmental sciences

For a reference copy of the document with all sections, see nature.com/documents/nr-reporting-summary-flat.pdf

Life sciences study design

All studies must disclose on these points even when the disclosure is negative.

Sample size	The sample size was determined based on the magnitude and consistency of measurable differences between groups (n = (2sigma^2)(Z_1-beta + Z_1-alpha/2)^2 / delta^2), and at least the minimal application size of relative statistical methods was used.
Data exclusions	For the hepatocyte primary culture, cells with inappropriate density, high mortality, and uneven distribution after seeding were excluded before experimental processing.<br>For the animal experiments, mice with unsuitable situations, such as huge change of body weight, mobility problem, distressed behavior, and fight wounds, were precluded.<br>For the tissue slices with uneven staining were removed.
Replication	All attempts of replication were gave similar results. At least 5 to 10 mice were used for in vivo studies for each group. Primary culture experiments were done at least triplicate, and all attempts at replication were successful.
Randomization	Mice and cultured hepatocytes were randomly assigned for time-course study and drugs treatment. Imaging fields were stochastically selected during image acquisition.
Blinding	Investigators were not blinded to drugs treatment during experiments. However, the investigators were blinded to group allocation during data collection, and the persons performing data analysis were unaware of the sample identity.

Reporting for specific materials, systems and methods

We require information from authors about some types of materials, experimental systems and methods used in many studies. Here, indicate whether each material, system or method listed is relevant to your study. If you are not sure if a list item applies to your research, read the appropriate section before selecting a response.

Materials & experimental systems

n/a	Involved in the study	
☐	☒	Antibodies
☒	☐	Eukaryotic cell lines
☒	☐	Palaeontology and archaeology
☐	☒	Animals and other organisms
☒	☐	Human research participants
☒	☐	Clinical data
☒	☐	Dual use research of concern

Methods

n/a	Involved in the study	
☒	☐	ChIP-seq
☐	☒	Flow cytometry
☒	☐	MRI-based neuroimaging

Antibodies

Antibodies used	Detailed information about the antibodies used in this study are shown as follows.<br>Antibodies used in detecting specific proteins are: BrdU (GeneTex, GTX26326, clone# BU1/75, 500x for IHC), GAPDH (Santa Cruz, sc-25778, clone# FL-335, 1000x for WB), β-actin (Sigma-Aldrich, A5441, clone# AC-15, 1000x for WB), α-tubulin (Sigma-Aldrich, T5168, clone# B-5-1-2, 1000x for WB), b-Catenin (Santa Cruz, sc-7199, clone# H-100, 500x for IHC), Glutamine Synthetase (BD, 610518, lot# 4357628, 1000x for IHC), AURKB (Abcam, ab2254, lot# GR3210135-1, 1000x for WB), pT232-AURKB (Rockland, 660-401-667, lot# 30691, 1000x for WB; Cell signaling, 2914, lot# 9, 1000x for WB), Histone H3 (ABclonal, A2348, lot# 350237701, 1000x for WB), pS10-Histone H3 (Cell Signaling, 9701, lot# 17, 1000x for WB), 9713),a-fetoprotein (Santa Cruz, sc-130302, clone# 39,

	lot# B1518, 250x for IHC), Ep-CAM (Santa Cruz, sc-53532, clone# G8.8, lot# H167, 250x for IHC), Lamin B1 (ABclonal, A1910, lot# 3560261001, 1000x for IHC), and gH2AX (ABclonal, AP0687, lot# 4000000110, 1000x for IHC).
Validation	All antibodies have been validated by companies with suitable controls, and reported by previous studies. The validation statements including relevant citations and antibody profile can be found on the websites of manufacturers which are showed as below.<br>BrdU (GeneTex, GTX26326, clone# BU1/75, 500x for IHC),<br>http://www.genetex.com/Web/Product/ProductToPDF.aspx?No=GTX26326&Country=46<br>GAPDH (Santa Cruz, sc-25778, clone# FL-335, 1000x for WB),<br>https://www.scbt.com/p/gapdh-antibody-fl-335<br>β-actin (Sigma-Aldrich, A5441, clone# AC-15, 1000x for WB),<br>https://www.sigmaaldrich.com/catalog/product/sigma/a5441?lang=en&region=TW<br>α-tubulin (Sigma-Aldrich, T5168, clone# B-5-1-2, 1000x for WB),<br>https://www.sigmaaldrich.com/catalog/search?term=t5168&interface=All&N=0<br>+&mode=partialmax&lang=en&region=TW&focus=product<br>b-Catenin (Santa Cruz, sc-7199, clone# H-100, 500x for IHC),<br>https://www.scbt.com/p/beta-catenin-antibody-h-102<br>Glutamine Synthetase (BD, 610518, lot# 4357628, 1000x for IHC),<br>https://www.fishersci.com/shop/products/anti-glutamine-synthetase-clone-6-bd-150-g-unlabeled/bdb610518<br>AURKB (Abcam, ab2254, lot# GR3210135-1, 1000x for WB),<br>https://www.abcam.com/aurora-b-antibody-ab2254.html<br>pT232-AURKB (Rockland, 660-401-667, lot# 30691, 1000x for WB; Cell signaling, 2914, lot# 9, 1000x for WB),<br>https://www.labome.com/product/Rockland-Immunochemicals/600-401-262.html<br>https://www.cellsignal.com/products/primary-antibodies/phospho-aurora-a-thr288-aurora-b-thr232-aurora-c-thr198-d13a11-xp-rabbit-mab/2914?Ntk=Products&Ntt=2914<br>Histone H3 (ABclonal, A2348, lot# 350237701, 1000x for WB),<br>https://abclonal.com/catalog-antibodies/HistoneH3RabbitpAb/A2348<br>pS10-Histone H3 (Cell Signaling, 9701, lot# 17, 1000x for WB), 9713)<br>https://www.cellsignal.com/products/primary-antibodies/phospho-histone-h3-ser10-antibody/9701?Ntk=Products&Ntt=9701<br>a-fetoprotein (Santa Cruz, sc-130302, clone# 39, lot# B1518, 250x for IHC),<br>https://www.scbt.com/p/afp-antibody-39<br>Ep-CAM (Santa Cruz, sc-53532, clone# G8.8, lot# H167, 250x for IHC),<br>https://www.scbt.com/p/ep-cam-antibody-g8-8?requestFrom=search<br>Lamin B1 (ABclonal, A1910, lot# 3560261001, 1000x for IHC),<br>https://abclonal.com/catalog-antibodies/LMNB1PolyclonalAntibody/A1910<br>gH2AX (ABclonal, AP0687, lot# 4000000110, 1000x for IHC)<br>https://abclonal.com/catalog-antibodies/PhosphoH2AXS139RabbitmAb/AP0687

Animals and other organisms

Policy information about studies involving animals; ARRIVE guidelines recommended for reporting animal research

Laboratory animals	All mice used for experiments were standard male ICR strain exception of special cases. Female NSG™ immunodeficient mice (The Jackson Laboratory, stock No: 005557) with 8 weeks age were conducted for subcutaneous implantation. For high-fat diet treatment, wild type C57BL/6J mice were utilized, which are susceptible to diet-induced obesity at 8 weeks age. Mice were maintained in 12h light/ 12h dark cycle (LD) with food and water ad libitum. Temperatures of 20-23°C with 40-60% humidity are maintained.
Wild animals	No wild animals were used in this study.
Field-collected samples	No field-collected samples were used in this study.
Ethics oversight	Animal studies were approved by the animal experimentation committee of Taipei Medical University and performed in accordance with the guidelines of the institutional committee for the use of animals for research. IACUC at TMU Approval No: LAC-20l6-0323.

Note that full information on the approval of the study protocol must also be provided in the manuscript.

Flow Cytometry

Plots

Confirm that:

☒ The axis labels state the marker and fluorochrome used (e.g. CD4-FITC).

☒ The axis scales are clearly visible. Include numbers along axes only for bottom left plot of group (a 'group' is an analysis of identical markers).

☒ All plots are contour plots with outliers or pseudocolor plots.

☒ A numerical value for number of cells or percentage (with statistics) is provided.

Methodology

Sample preparation	As indicated in Supplementary material, hepatocytes were isolated by collagenase perfusion system and resuspended in ice-cold PBS at a density of 2 x 10^6 cells/ml. Cells were fixed with 4% PFA at 4°C overnight with gentle rotation. Cells were then washed with PBS containing 0.25 mg/ml RNase A and stained with 2 ug/ml DAPI at 4°C for 60 min. DNA content of labeled

	cells was measured by flow cytometer.
Instrument	DNA content of labeled cells was measured by flow cytometer (Becton Dickinson, LSRII SORP – 17 color analyzer).
Software	Acquired data were analyzed by BD FACSDiva software v6.2. and FlowJo 7.6.1.
Cell population abundance	The distribution of polyploid hepatocytes is consistent with our IHC data, which displays higher polyploidy in centrilobular region than in perilobular region of the liver after DEN treatment.
Gating strategy	Cells were sorted by FSC and SSC to eliminate cell debris, then sorted for singlet cell by DAPI-A and pulse width.

☒ Tick this box to confirm that a figure exemplifying the gating strategy is provided in the Supplementary Information.
