# Supplementary figures and images for: Hyperpolyploidization of hepatocyte initiates preneoplastic lesion formation in the liver
Source: Nat Commun. 2021 Jan 28;12:645. doi: 10.1038/s41467-020-20572-8 (PMC7844417; doi:10.1038/s41467-020-20572-8)

Fig. 4d

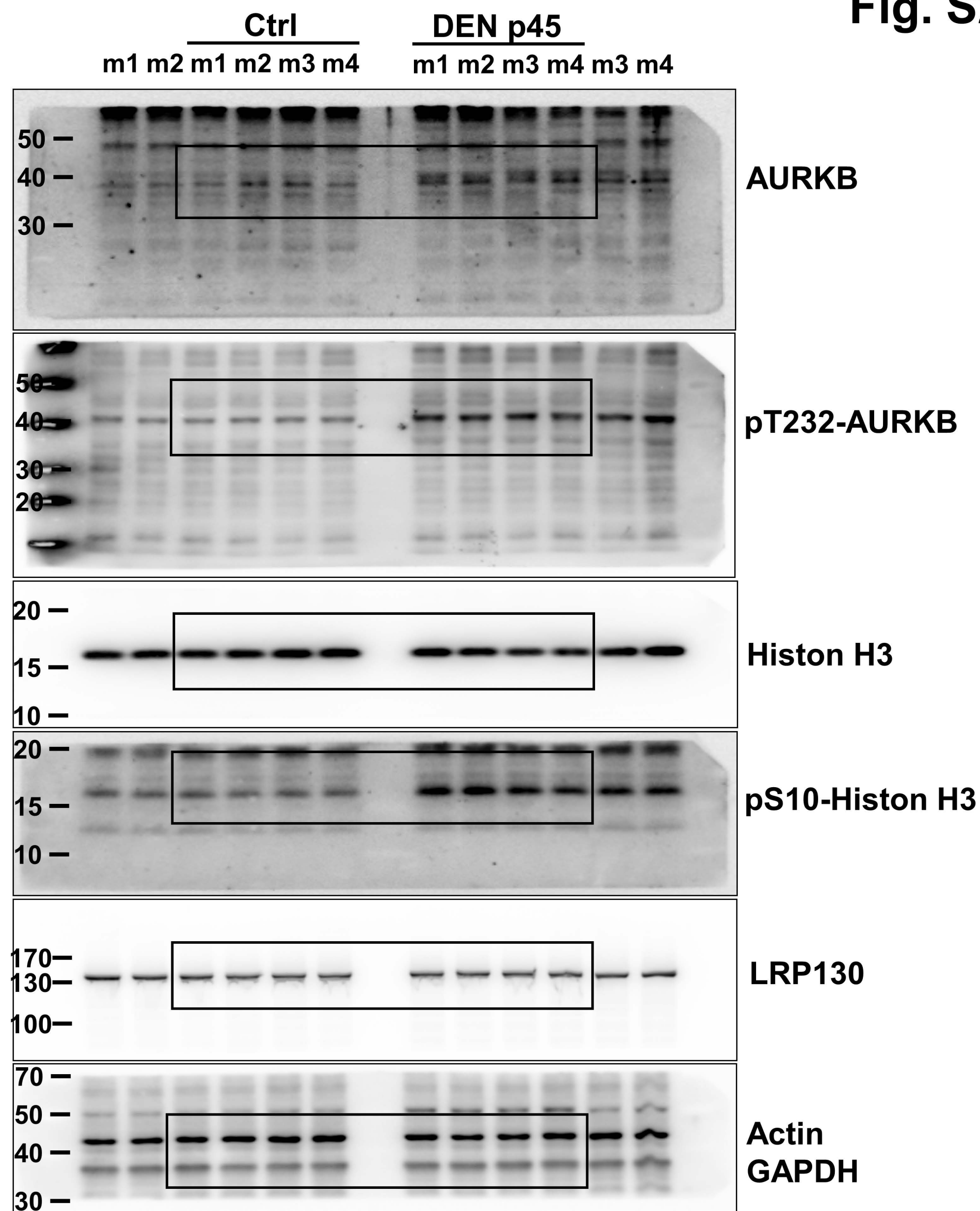

Fig. S2f

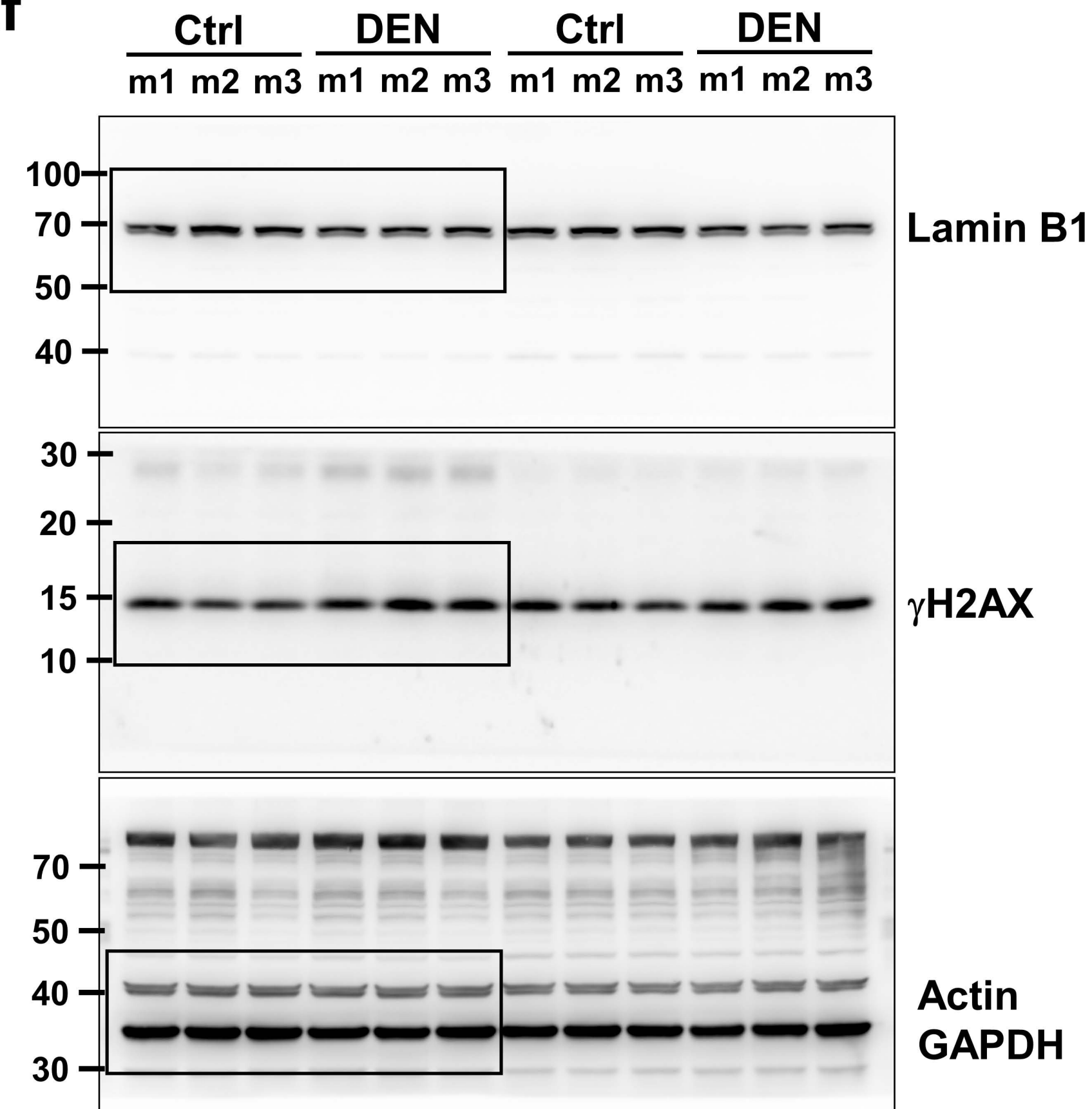

Fig. S5e

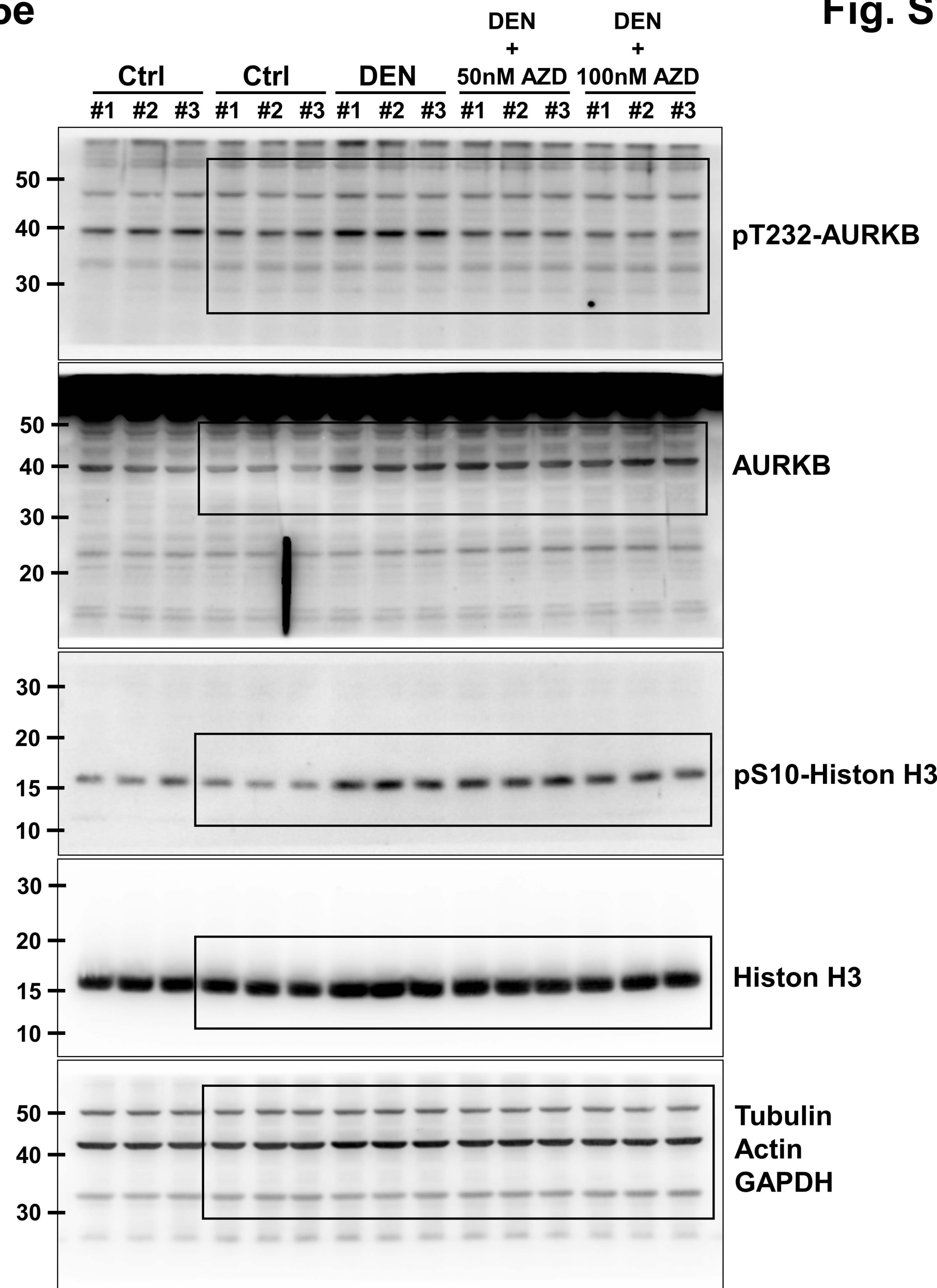

Fig. S7b

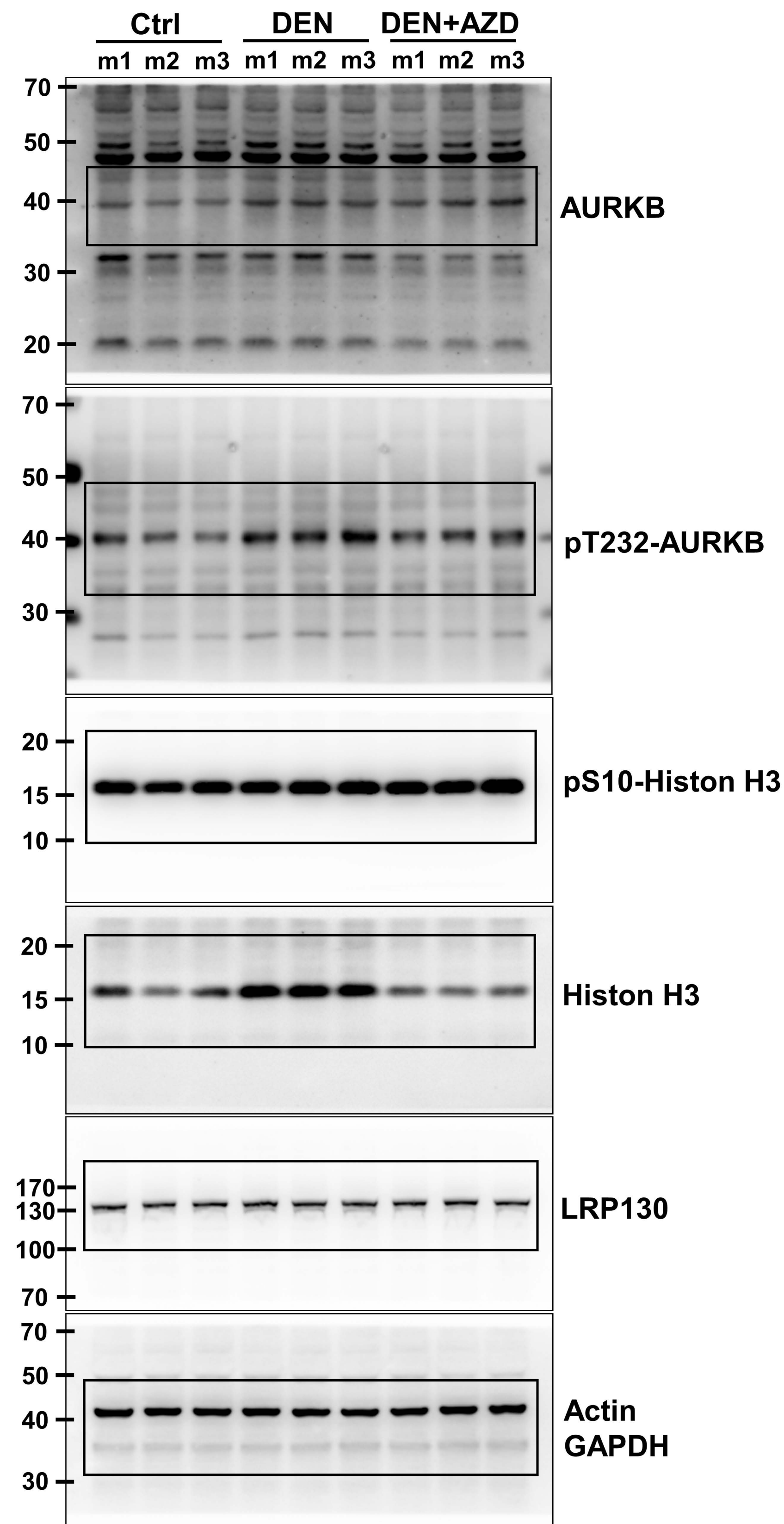

Supplement: Supplementary file 12 — Source Data [file 41467_2020_20572_MOESM12_ESM.zip › Source Data/20201105 Source Data-uncrop blots-300dpi.pdf]
